# Metabolic modeling reveals determinants of synbiotic efficacy in a human intervention trial

**DOI:** 10.1101/2025.06.24.25330246

**Published:** 2025-06-25

**Authors:** Nick Quinn-Bohmann, Sean M. Gibbons

**Affiliations:** 1 Institute for Systems Biology, Seattle, WA 98109, USA;; 2 Bioengineering Department, University of Washington, Seattle, WA 98195, USA;; 3 Genome Sciences Department, University of Washington, Seattle, WA 98195, USA;; 4 eScience Institute, University of Washington, Seattle, WA 98195, USA;

## Abstract

Synbiotic interventions show variable effects across individuals, likely driven by ecological interactions with the endogenous microbiota and the host diet. Rationally predicting individual-specific success or failure of probiotic and prebiotic interventions remains an outstanding challenge. In this study, we leverage microbial community-scale metabolic models (MCMMs) to predict probiotic engraftment and shifts in microbiota-mediated short-chain fatty acid (SCFA) production in response to a synbiotic intervention. Using data from a placebo-controlled synbiotic intervention trial, involving a cocktail of five probiotic strains and the prebiotic inulin, we validate model engraftment predictions with quantitative PCR (qPCR), demonstrating that MCMMs accurately predict probiotic engraftment outcomes in the treatment group with over 85% accuracy. Engraftment varied by species, with *Akkermansia muciniphila* and *Bifidobacterium infantis* displaying higher engraftment rates than *Clostridium beijerinckii, Anaerobutyricum hallii,* and *Clostridium butyricum*. Furthermore, MCMMs predicted significant increases in butyrate and propionate production following synbiotic treatment. MCMM-predicted changes in propionate production in the treatment group were negatively associated with changes in C-reactive protein (CRP), a blood marker of systemic inflammation, from baseline to 12 weeks after the synbiotic intervention. Finally, we explore MCMM-predicted responses to a wider range of synbiotic combinations in a larger observational cohort, suggesting that personalized prebiotic selection can augment probiotic efficacy. These findings highlight the potential of metabolic modeling to inform precision microbiome therapeutics.

## Introduction

Synbiotics, which combine probiotic microbes with the compounds that these microbes like to eat (i.e., prebiotics), represent a promising avenue for improving the efficacy of microbiome-mediated interventions, targeting everything from prediabetes and autoimmune disorders to neurodegenerative diseases^1–3^. The human colonic microbiota plays a critical role in regulating host physiology and immunity^4–8^. Probiotic and prebiotic administration have been shown to influence the metabolic outputs of the endogenous microbiota, inhibit pathogen colonization, and enhance mucosal barrier integrity^9,10^. In addition to immunomodulation and supporting metabolic health, emerging evidence suggests that synbiotic interventions may even impact brain function via the gut-brain axis^11–13^.

Despite these promising findings, the efficacy of probiotic, prebiotic, and synbiotic interventions can vary significantly between individuals, posing a major challenge for their widespread clinical application^14,15^. Engraftment of a probiotic strain—the ability of the administered microbe to establish and persist within the gut ecosystem—depends on multiple factors, including the availability of a suitable metabolic niche, competitive and cooperative interactions with endogenous microbiota, and interactions with the host immune system^16,17^. Beyond the composition of the commensal microbiota, the interplay between factors like total commensal biomass, propagule pressure (i.e., the probiotic dose), available prebiotic substrates, and host diet can all impact engraftment^18^. In order to rationally predict probiotic engraftment and prebiotic effects across individuals and dietary backgrounds, we require methods that integrate this complexity.

Genome-scale metabolic models (GEMs) provide a powerful mechanistic framework for estimating microbial growth and metabolism^19,20^. Recent advances in constraint-based modeling have extended this approach to diverse microbial communities, yielding microbial community-scale metabolic models (MCMMs)^21^. By integrating genome-scale metabolic reconstructions from hundreds of gut bacterial taxa with flux balance analysis, MCMMs enable predictions of microbe-microbe interactions, competition for resources, and ecosystem-scale metabolic behaviors^21,22^. These models have demonstrated predictive accuracy in estimating microbial growth rates, short-chain fatty acid production, methane production, and metabolic shifts in response to disease status or dietary interventions^21–25^. Notably, prior work has shown that MCMMs can capture the engraftment potential of opportunistic pathogens, such as *Clostridioides difficile,* suggesting that similar approaches could be used to model probiotic engraftment in individual hosts^22,23^.

To validate this approach, we aimed to use MCMMs to predict the enrichment of probiotic species based on data from a human intervention trial conducted by Perradeau et al. (2020)^26^. In this double-blind, placebo-controlled intervention trial, participants previously diagnosed with type 2 diabetes mellitus were treated for 12 weeks with either a placebo or a multi-strain synbiotic cocktail (WBF-011). This formulation contained *Akkermansia muciniphila* (AMUC), *Anaerobutyricum hallii* (AHAL), *Bifidobacterium infantis* (BINF), *Clostridium beijerinckii* (CBEI), and *Clostridium butyricum* (CBUT), supplemented with a low-dose of inulin (0.3g), a prebiotic fiber known to support probiotic growth. Participants who received the synbiotic exhibited a significantly greater reduction in the area under the curve (AUC) for glucose during a standard glucose tolerance test from Week 0 to Week 12, compared to the placebo group, indicating a beneficial population-level effect on glycemic control^26^.

Using metagenomic sequencing date, quantitative PCR (qPCR), and immune and metabolic profiling, we assessed the ability of MCMMs to accurately predict probiotic engraftment and the subsequent impact on host metabolism and immune function. Specifically, we evaluated how well MCMM-derived predictions align with observed shifts in microbial composition, metabolite production, and inflammatory markers. Finally, we expand MCMM simulations to a cross-sectional cohort of 156 generally-healthy individuals, to identify personalized prebiotic, dietary, and probiotic interventions that are predicted to maximize individual-specific efficacies of synbiotic interventions. Together, these results provide a promising computational platform for designing personalized synbiotics for human intervention trials.

## Results

### qPCR shows enrichment of probiotic species in the treatment group

Custom qPCR primer pairs were designed for each of the five probiotic strains in the WBF-011 treatment^26^. Cycle threshold (Ct) values were obtained for all five species in each sample, and were inverted (1/Ct) to serve as a proxy for species abundance. Samples were collected at multiple time points throughout the study, including at baseline (Week 0), during treatment (Week 4), and at the end of treatment (Week 12).

**Figure 1.**
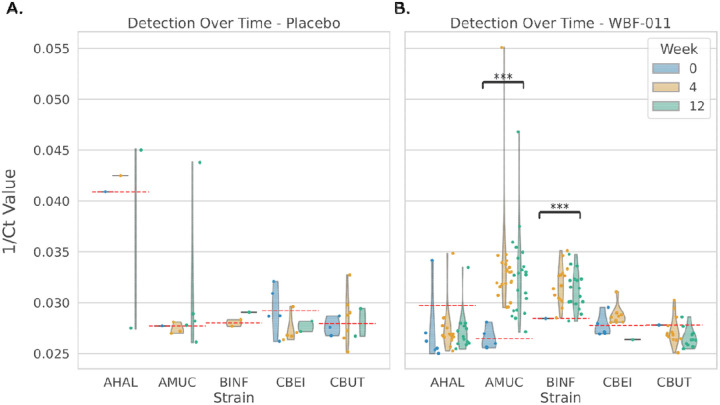
qPCR quantification of probiotic strain abundances in WBF-011 and placebo arms. (A) There were no significant enrichments in probiotic strains over time in the placebo treatment group, although a few samples showed high 1/Ct values (likely due to primer binding to phylogenetically related endogenous strains). (B) There was a significant enrichment in AMUC and BINF probiotics in the WBF-011 treatment arm. There was no enrichment of CBEI, CBUT and AHAL probiotics in the WBF-011 treatment arm. Significant increase determined by Mann-Whitney U test, *** = p < 0.001.

In the placebo group, there were no significant changes in 1/Ct values over time for any of the five probiotic strains (Fig. 1A). In the WBF-011 treatment group, AMUC and BINF exhibited significant enrichment from Week 0 to Week 12 across most participants (Fig. 1B, Mann-Whitney U test, AMUC: p = 2.7*10^−7^; BINF: p = 4.7*10^−7^). At Week 12, the mean 1/Ct for AMUC was 0.033 ± 0.0009, exceeding the species-specific detection threshold of 0.0264, while the mean 1/Ct for BINF was 0.031 ± 0.0004, surpassing its detection threshold of 0.0284. In contrast, CBEI, AHAL, and CBUT showed no comparable enrichment. In nearly all samples, 1/Ct values for these species either remained unchanged or declined after 12 weeks. Generally, these trends suggest that AMUC and BINF probiotics were able to engraft in the majority of individuals included in this study. In the placebo group, enrichment was observed in some samples, though these occurrences were sparse and may have been driven by off-target primer binding to closely related endogenous strains (Fig. 1A).

### MCMMs predict species-level enrichment

Models were constructed using Week 0 metagenomic sequencing data from participants in the WBF-011 treatment arm to assess whether MCMMs could predict the engraftment patterns observed in the qPCR results (Fig. 1). For each individual, simulations were run to model the addition of the five probiotic species, assuming a standard European diet, and predicted growth rates were obtained. Growth rates were binarized at a threshold of 0.001 h^−1^, above which represented growth and below which represented non-growth, and these binary classifications were compared to qPCR-based engraftment thresholds, described above.

**Figure 2:**
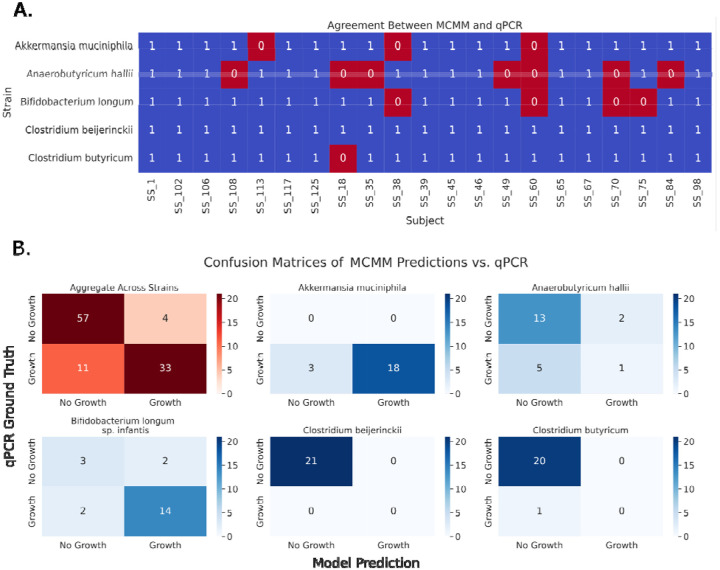
MCMM predictions for probiotic growth show significant agreement with qPCR engraftment thresholds. . (A) MCMM-predicted growth rates binarized around a threshold of 0.001 agree with qPCR detection thresholds in 90 out of 105 observations (Cohen’s k = 0.70, indicating substantial agreement). Blue boxes containing a “1” indicate agreement, red boxes containing a “0” indicate disagreement between the model and the qPCR data. (B) Confusion matrices describing results across and within probiotic strains, showing highly significant agreement (Fisher’s Exact Test, aggregate results across all strains, p = 1.3×10^−13^).

Across all comparisons (5 predictions for each of 21 samples, total N = 105), model-predicted growth and qPCR results showed 85.7% agreement (Fig. 2A, Cohen’s k = 0.70, indicating substantial agreement). Perfect agreement was observed in 11 of 21 participants, where all five probiotic engraftment statuses were accurately predicted. Additionally, CBEI was correctly predicted as not growing for all 21 participants, while AHAL had the lowest accuracy, with misclassification in 7 of 21 cases. Overall, there was significant concordance between predictions and observations (Fig. 2B). Across all strains, 57 instances of growth were correctly predicted, 33 instances of non-growth were correctly predicted, 4 cases were incorrectly predicted as growth, and 11 cases were incorrectly predicted as non-growth (Fisher’s exact test, p = 1.3 × 10^−13^).

### Prediction of Microbial Short-Chain Fatty Acid Production

To evaluate the predicted functional impact of WBF-011 administration, we analyzed the model-predicted production of butyrate and propionate, two key short-chain fatty acids with well-documented metabolic health-promoting, anti-inflammatory, and gut barrier-strengthening properties^27^. Prior work has demonstrated the accuracy of MCMM-based predictions for SCFA production in the human gut microbiome^22^, providing a strong rationale for employing this approach to assess the mechanistic contributions of both the probiotic formulation (AMUC, AHAL, BINF, CBEI, and CBUT) and the prebiotic (inulin) used in this study.

**Figure 3.**
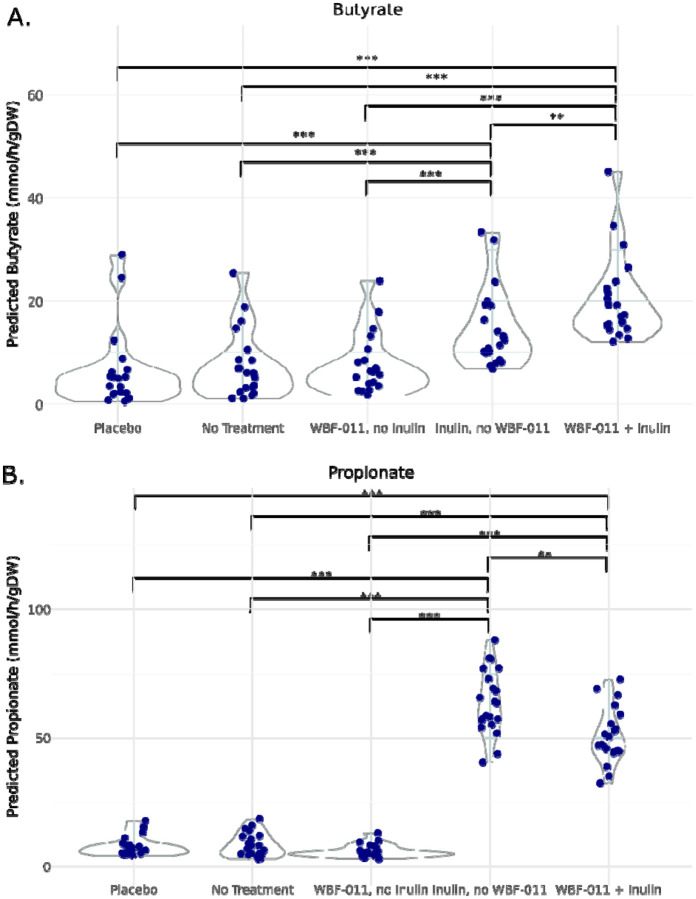
MCMM–predicted SCFA production rates shift in response to prebiotic and probiotic treatment. (A) Butyrate production in MCMM simulations increased in response to prebiotic and synbiotic supplementation. Significant increases were observed with inulin alone, as well as with the combined inulin-WBF-011 treatment. (B) Propionate production in MCMM simulations also increased with inulin and synbiotic treatment, relative to no treatment. However, propionate production declined in the synbiotic condition, relative to the prebiotic-only condition. Each point represents the predicted production level fora single sample. **: p < 0.01, ***: p < 0.001, Mann-Whitney U test.

To systematically dissect the influence of probiotic and prebiotic interventions on SCFA production, we conducted five distinct MCMM simulations on a standard European dietary background: (1) microbiomes from placebo group participants, (2) treatment group microbiomes without any probiotic or prebiotic addition, (3) treatment group microbiomes with probiotic supplementation alone, (4) treatment group microbiomes with prebiotic supplementation alone, and (5) treatment group microbiomes with combined synbiotic supplementation. Following simulation, total MCMM-predicted butyrate and propionate production rates were compared across conditions (Fig. 3).

As expected, both the placebo group and the no treatment group exhibited relatively low levels of butyrate production (Fig. 3A, Placebo: 6.59 ± 1.89 mmol/gDW/h; No Treatment: 7.53 ± 1.41 mmol/gDW/h). The addition of the WBF-011 probiotic cocktail in the absence of prebiotic supplementation did not significantly alter butyrate production (7.62 ± 1.24 mmol/gDW/h), although there was an increasing trend. In contrast, treatment group simulations incorporating inulin, either alone (14.7 ± 1.64 mmol/gDW/h) or in combination with the probiotic cocktail (20.4 ± 1.78 mmol/gDW/h), resulted in significantly elevated butyrate production compared to the first three groups (Mann-Whitney U test, p < 0.05). Importantly, butyrate production was also significantly higher in the combined synbiotic condition relative to prebiotic supplementation alone (Mann-Whitney U test, U = 333.0, p = 0.0048), indicating a synergistic interaction between inulin and the probiotic strains.

Similar results were observed for propionate. The placebo, no treatment, and probiotic only group showed low levels of propionate production (Fig. 3B, Placebo: 8.03 ± 0.82 mmol/gDW/h; No Treatment: 8.42 ± 0.99 mmol/gDW/h; Probiotic Only: 6.07 ± 0.53 mmol/gDW/h). The addition of prebiotic substrate alone or in combination with the probiotic cocktail significantly increased the production of propionate (Prebiotic Only: 63.82 ± 2.72 mmol/gDW/h), Prebiotic + Probiotic: 50.67 ± 2.32 mmol/gDW/h, Mann-Whitney U test, p < 0.05). However, in this case, there was a significant decrease in propionate production in the combined prebiotic-probiotic condition as compared to the prebiotic condition alone (Mann-Whitney U test, U = 93.0, p = 0.0014). These significant increases in MCMM-predicted butyrate and propionate production rates may help to explain the significant decrease in glucose AUC observed in the WBF-011 treatment group, relative to the placebo^26^.

### C-Reactive Protein Associates with Predicted SCFA Production

In Perraudeau et al.^26^, C-reactive protein (CRP), a marker of systemic inflammation, was a primary endpoint measure. In the original study, there were no significant differences in average CRP levels between the treatment and placebo groups. Previous studies have shown that MCMM-based predictions of SCFA production from the gut microbiome show significant associations with the host inflammation state, in line with the known anti-inflammatory effect of these metabolites^22^. We compared the predicted levels of butyrate and propionate production from samples in the treatment group, receiving the combined synbiotic cocktail, against changes in serum CRP levels from baseline to the study endpoint. To determine the overall treatment effect, change in CRP from Week 0 to Week 12 (ΔCRP) was compared against the change in butyrate or propionate production from pre-treatment to post-treatment (Δbutyrate and Δpropionate, respectively).

**Figure 4.**
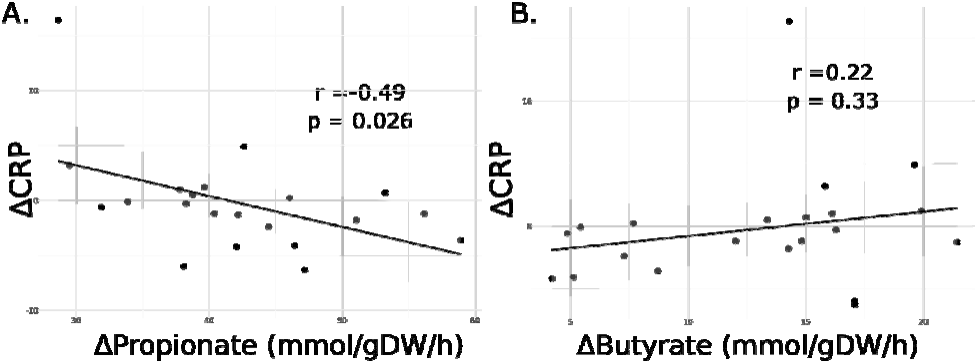
Post-intervention changes in inflammatory marker CRP shows association with MCMM-predicted changes in propionate, but not butyrate, production rates relative to baseline in the synbiotic treatment group. (A) ΔCRP showed significant negative association with MCMM-predicted Δpropionate in models treated with the synbiotic (linear regression, r = −0.49, p = 0.026, t-test for slope). (B) MCMM-predicted Abutyrate did not show a significant association with ΔCRP (linear regression, r = 0.22, p = 0.33, t-test for slope).

Δpropionate showed a significant negative association with ΔCRP within the treatment group (Fig. 4A, linear regression, r = −0.48, p = 0.025). However, the association between Δbutyrate and ΔCRP did not reach significance (Fig. 4B, linear regression, r = 0.22, p = 0.33).

### Personalized MCMM-Predicted Engraftment and SCFA Production Predictions for a Probiotic Cocktail Across Different Prebiotic and Dietary Backgrounds in a Large Observational Cohort

We ran MCMM simulations in a cohort of 156 individuals from a former precision wellness company (Arivale) with newly-generated stool metagenomic sequencing data, balanced for age and sex. To demonstrate the broader applicability of this methodology in designing personalized synbiotics, growth of WBF-011 strains was simulated across six prebiotic and dietary contexts: (1) a standard European diet without prebiotic supplementation, (2) a standard European diet supplemented with inulin, (3) a standard European diet supplemented with pectin, (4) a standard European diet supplemented with resistant starch, (5) a standard European diet supplemented with maltodextrin, (6) a standard European diet supplemented with hemp seed, (7) a standard European diet supplemented with psyllium husk, and (8) a standard high-fiber diet.

**Figure 5.**
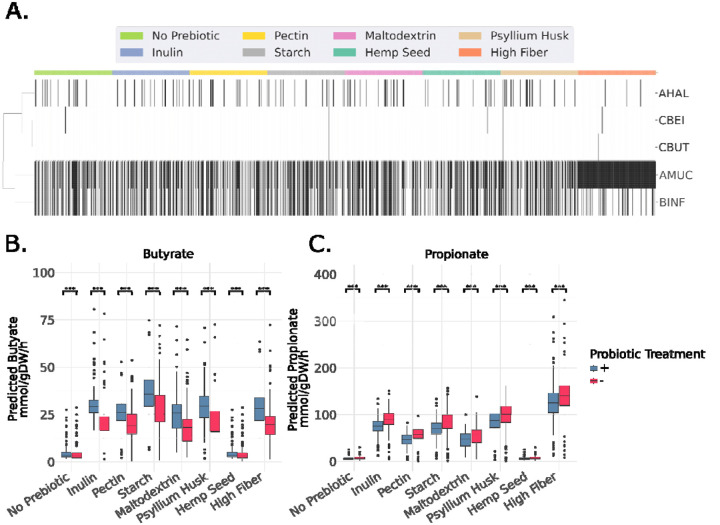
MCMM-predicted engraftment and SCFA production rates across simulated probiotic and prebiotic interventions in a cohort of generally-healthy Americans. (A) Using a cohort of 156 individuals, we predicted engraftment, using a growth rate binarization threshold of 10^−3^. Differential engraftment was observed across prebiotic and dietary backgrounds. Black cells denote engraftment, while white cells indicate non-engraftment. (B-C) Butyrate and propionate production rates were increased upon treatment with prebiotic substrates on a standard European diet (inulin, pectin, resistant starch, maltodextrin, psyllium or hemp seed), or with a shift from a standard European to a standard high-fiber diet. Probiotics tended to increase butyrate production rates and decrease propionate production rates, relative to the prebiotic-alone treatments. Color encoding demonstrates the effect of adding a probiotic cocktail to the prebiotic/dietary treatments. Significance determined by Mann-Whitney U-test, *** = p<0.001.

Probiotic engraftment predictions varied across prebiotic and dietary backgrounds (Fig. 5A). For instance, the switch from a standard European diet to a high-fiber diet resulted in a much higher average engraftment rate for AMUC (growth in 100% of samples, versus 57% on the standard European diet), but a decrease in engraftment for BINF (growth in 17% of samples, versus 42% on the standard European diet). MCMM-predicted butyrate and propionate production rates increased significantly as a result of both prebiotic and probiotic addition (Fig. 5B–C). The addition of probiotic consortia showed significant increases in predicted butyrate production in every case, except for when no prebiotic substrate was added (Mann-Whitney U test, p < 0.05). For propionate, a significant decrease in predicted production was observed in every case (Mann-Whitney U test, p < 0.05). A shift away from a standard European diet to a high-fiber diet showed the largest increase in both predicted butyrate and propionate production rates (Fig. 5B–C, Butyrate: Mann-Whitney U test, U = 251, p = 1.4×10^−50^; Propionate: Mann-Whitney U test, U = 4.0, p = 1.3×10^−52^).

**Figure 6.**
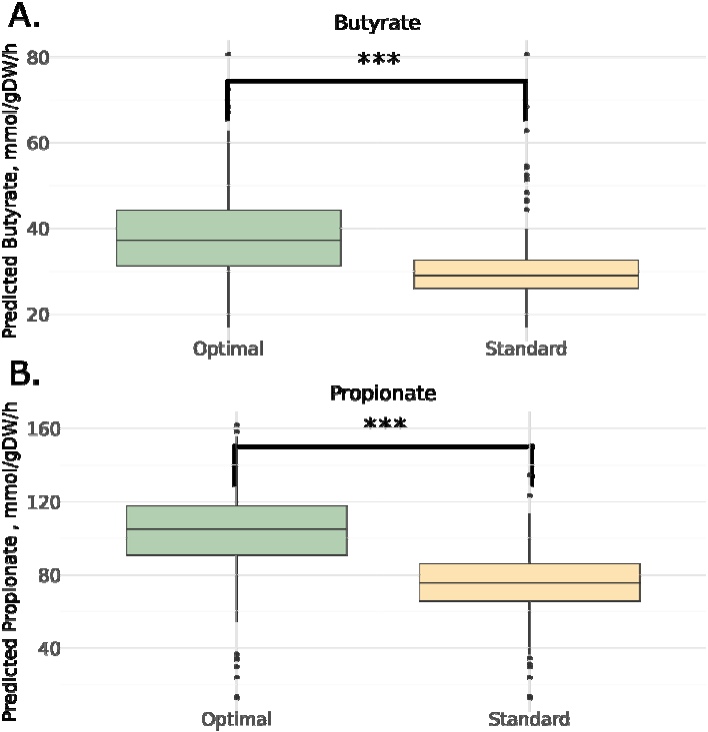
Personalized treatments outperform standard of care. For both SCFAs, an optimal prebiotic and/or probiotic treatment combination was selected and compared against a standard treatment, which consisted of inulin and the 5-strain probiotic cocktail. In both cases, the individual-specific optimal treatment showed significantly higher levels of production. Significance determined by Mann-Whitney U-test, *** = p<0.001.

For each individual, the optimal prebiotic-probiotic combination on the standard European diet was identified, in terms of maximizing predicted butyrate or propionate production rates. While some patterns emerged, the optimal intervention varied across individuals (Figs. S1–2). For butyrate production, the most common optimal treatment was the use of starch in combination with the 5-strain probiotic cocktail. For propionate production, psyllium husk with no probiotic cocktail was the most common optimal intervention. Overall, a personalized optimal solution resulted in significantly higher predicted levels of propionate and butyrate production when compared with the ‘standard of care’ synbiotic treatment, which was defined as combining inulin with the probiotic cocktail, as in the original WB-011 trial (Fig. 6).

## Discussion

The ability of an orally administered probiotic to successfully engraft in the gut is influenced by multiple ecological and host-specific factors, including the pre-existing composition of the gut microbiome and the background diet. A method for the rational prediction of probiotic engraftment and health-relevant microbial metabolite production, in the context of a given microbiota and diet, would open up new avenues for precision synbiotic design. Prior work by our group has demonstrated that our microbial community-scale metabolic modeling (MCMM) platform can be leveraged to predict personalized SCFA production rates and personalized pathobiont engraftment risk^22,23^. In order to validate our MCMM methodology in the context of synbiotic design, we leverage qPCR data from the WBF-011 synbiotic trial to determine whether or not we can accurately predict relevant outcomes. The WBF-011 trial showed variable engraftment rates across the five tested probiotic organisms: *Akkermansia muciniphila* (AMUC), *Bifidobacterium infantis* (BINF), *Clostridium beijerinckii* (CBEI), *Anaerobutyricum hallii* (AHAL), and *Clostridium butyricum* (CBUT). AMUC and BINF showed engraftment across most of the participants, while CBEI, AFIAL, and CBUT rarely engrafted. Given this heterogeneity, these trial data represented an ideal validation-case for our MCMM predictions.

Despite the inherent complexity of predicting probiotic growth, the MCMM-based predictive framework demonstrated substantial accuracy, showing over 85% agreement with observed probiotic engraftment patterns (Fig. 2A). These models incorporate key ecological determinants, like endogenous microbiome composition and the availability of prebiotic and dietary substrates, to estimate probiotic growth rates, which are closely aligned with experimental qPCR data. Notably, of the 105 total comparisons, 90 were accurately predicted, with 57 instances correctly classified as non-growth and 33 correctly classified as growth (Fig. 2B). This high level of accuracy in engraftment prediction was achieved in spite of the fact that we lacked personalized constraints on dietary intake (i.e., a standard European diet was applied to each model).

A fundamental goal of probiotic and prebiotic supplementation is to modulate the production of key microbial metabolites that support host health. Among these, short-chain fatty acids (SCFAs)—such as butyrate and propionate—are particularly significant due to their roles in maintaining gut barrier integrity, regulating proper immune function, and supporting metabolic homeostasis^22,27^. Synbiotic administration provides a means of enhancing SCFA production, and MCMMs can be leveraged to predict these metabolic shifts. As anticipated, MCMMs predicted a significant increase in the total production of butyrate and propionate in response to inulin supplementation, consistent with its role as a fermentable dietary fiber that serves as an ideal substrate for SCFA biosynthesis (Fig. 3). Notably, our analysis also revealed a synergistic effect between prebiotic administration and the WBF-011 probiotic cocktail. While inulin alone significantly increased butyrate production compared to baseline, the combination of inulin with WBF-011 led to an even greater increase (Fig. 3A). This finding aligns with prior studies highlighting the contributions of both *Bifidobacterium longum* and *Akkermansia muciniphila,* key members of the WBF-011 consortium, to butyrate production through cross-feeding interactions within the gut microbiota^28–30^. In contrast, both inulin alone and the prebiotic-probiotic combination increased propionate levels relative to the baseline, but the synbiotic combination resulted in significantly lower propionate production compared to the prebiotic alone (Fig. 3B). These results suggest that this probiotic cocktail may bias SCFA production away from propionate towards butyrate, shifting the metabolic output of a community. Such metabolic trade-offs are well-documented in microbial ecosystems, where competition for substrates influences the balance of SCFA production^21,22,27,31^.

Next, we sought to identify associations between synbiotic-induced changes in predicted SCFA production and the primary outcome variables. Perraudeau et al. identified a significant drop in the area under the curve (AUC) for postprandial glucose levels during a standardized meal-tolerance test over the 12-week trial period in the treatment group, but not in the placebo group^26^. This drop in glucose AUC mirrors the significant increase we found in MCMM-predicted butyrate and propionate production rates in response to the symbiotic treatment (Fig. 3). Given that increased intestinal SCFA availability has been linked to improved glycemic control^32^, the observed metabolic effects in this study may be partially attributable to the enhanced SCFA production induced by the WBF-011 and inulin combination^33^. Future intervention studies could further refine these insights by integrating longitudinal metabolomic and host-response data to establish more direct mechanistic links between microbial SCFA production and host metabolic outcomes.

CRP was the primary immunological endpoint measured during the WBF-011 trial, as it is a well established metric of systemic inflammation^34^. Based on collected patient data, no significant difference was observed in endpoint CRP measures between the treatment and placebo arms of the study. However, we observed that within the treatment arm, there was a significant negative association between the change in CRP and the model-predicted change in propionate production from Week 0 to Week 12 (Fig 4A). This relationship was not observed for butyrate. While butyrate is generally thought to be a more potent anti-inflammatory SCFA, propionate can also reduce inflammation^35^ and prior work from our group has observed a significant negative relationship between diet-mediated inflammation and MCMM-predicted propionate production^22^.

Given the strong engraftment validation we observed in the WBF-011 trial, we decided to expand our MCMM simulations to a larger cohort where we could explore engraftment and SCFA production predictions across a wider range of probiotics, prebiotics, and dietary backgrounds. Simulation results from a cohort of 156 generally-healthy individuals showed that variation in prebiotic and dietary inputs could substantially alter engraftment outcomes across individuals. We observed that a switch from the standard European diet to a high-fiber diet greatly improved the engraftment success of *Akkermansia muciniphila,* a probiotic shown to improve post-prandial glucose responses^26^. Additionally, microbial production of butyrate and propionate increased with prebiotic supplementation and with a switch to a standard high fiber diet. Not all individuals responded to the same prebiotic fibers, and there was a heterogenous response in terms of the most optimal treatment for each individual, indicating how MCMMs could be a valuable tool for optimizing prebiotic and probiotic pairings. Ultimately, this methodology could be leveraged to improve the administration of probiotics and prebiotics, depending on the baseline gut microbiome composition and the dietary background of the host.

During the course of our work on this project, we found that the single-strain species-level AGORA1 GEM for *Akkermansia muciniphila* failed to predict growth in any sample following its introduction, whereas the multi-strain species-level AGORA2 GEM successfully predicted growth consistent with experimental results. For most other commensal species in our simulations, we had multiple AGORA1 representative strains that could be included in a pan-strain model^36^. We hypothesize that the improvement in *Akkermansia* engraftment predictions between AGORA1 and AGORA2 stems from the inclusion of four *A. muciniphila* strains in AGORA2, compared to only a single strain in AGORA1. Given that individual strain-level models are often imperfectly curated, greater strain-level representation in species-level GEMS likely captures a broader range of metabolic capabilities, which appears to lead to better agreement between model predictions and experimental outcomes. While constructing GEMs for all possible strains remains impractical, increasing strain diversity within species-level models appears to offer clear benefits in these community-scale simulations. Interestingly, this phenomenon may be rooted in the biology of real-world ecosystems, where we often observe the coexistence of 2–3 strains for each bacterial species in the human gut^37^.

Despite promising results, there are certain limitations implicit to the methodology presented here. Principal among these is that existing GEM databases do not include all extant strains for a given gut bacterial species. The consequence of this is that MCMM-based analyses such as these are limited to species-level resolution, using models that summarize the metabolic capacity of available strains within a given species. For a trial such as this, strain-specific engraftment cannot be isolated from growth predictions of endogenous strains. For this reason, we centered our engraftment definition on enrichment of a species above its baseline level following treatment. Future work should focus on accounting for strain-level differences in metabolic capacity that differ between probiotic and endogenous strains of a given species. Another limitation of this analysis was the lack of participant dietary intake data. Better constraints on personalized dietary intake should further improve the predictive accuracy that we observed in the current analysis, which assumed that everyone was eating the standard European diet. Finally, the current methodology for constructing and solving MCMMs does not account for spatial structure or host physiology within the gut. For instance, *Akkermansia muciniphila* typically grows within the mucus layer of the colonic epithelium, in a different microenvironment than many other taxa within the microbiota. For this reason, it is more closely tied to the colonic epithelium, and the production of host-derived compounds. Future work should aim to integrate MCMMs with metabolic models of the colonic epithelium^38^, which may help to further improve predictions.

Taken together, these findings demonstrate the utility of MCMMs as a predictive framework for assessing synbiotic interventions at the individual and population levels. By integrating metabolic modeling with experimental validation, we highlight key ecological and metabolic determinants of probiotic engraftment success and SCFA production, providing a pathway for more targeted microbiome-based therapies. However, realizing the full potential of these models will require continued refinement, including improved strain-level resolution, more comprehensive dietary intake data, and enhanced host-microbiome interaction modeling. Future research should aim to bridge these gaps by incorporating multi-omics approaches, longitudinal sampling, and personalized dietary profiling. Ultimately, leveraging MCMMs in a clinical setting could enable precision microbiome therapeutics tailored to an individual’s metabolic and inflammatory profile, optimizing probiotic and prebiotic efficacy across diverse patient populations.

## Methods

### Data collection

Validation data used in this study were obtained from Perraudeau et al. (2020). The study cohort consisted of 76 participants previously diagnosed with type 2 diabetes. Participants were randomized into two experimental groups (N = 27 and N = 23) and a placebo group (N = 26). 10, 6 and 2 participants were lost to follow-up in each study arm, respectively. Those in the experimental groups received one of two probiotic formulations, WBF-010 or WBF-011. WBF-010 contained strains of *Bifidobacterium longum subsp. infantis, Clostridium butyricum,* and *Clostridium beijerinckii*. WBF-011 included the same strains as WBF-010 with the addition of *Akkermansia muciniphila* and *Anaerobutyricum hallii*. Both probiotic formulations were supplemented with a small amount of inulin (0.3 grams), a prebiotic derived from chicory root. Participants in all three study arms followed the intervention for 12 weeks, consuming three capsules twice daily. The primary metabolic endpoint assessed in the original trial was the change in the area under the curve (AUC) for glucose during a standard three-hour meal-tolerance test (MTT) conducted at baseline and after 12 weeks. The ΔAUC values were calculated by subtracting baseline AUC from the 12-week AUC. Analysis revealed that WBF-011, but not WBF-010, led to a significant shift in AAUC compared to the placebo, so only the WBF-011 study arm was used for downstream microbial community-scale metabolic model (MCMM) predictions.

Additional data used in the analysis were collected from Arivale, Inc. (Seattle, WA). Arivale closed its operations in 2019. De-identified metagenomic data from patient-collected fecal samples was collected and used to construct MCMMs as described below. Participants included in the analysis (N = 156) were adults in the United States (92 female, 64 male sex assigned at birth), with average age 47.4 ± 1.0 years. Metagenomes are available on the NCBI Sequence Read Archive under accession number PRJNA1262070.

### Model Construction

Metagenomic sequencing was performed on stool samples collected at baseline and after 12 weeks. Raw sequencing reads were processed using Kraken2 (v1.01) for taxonomic classification, and Bracken was used to refine taxonomic abundance estimates^39,40^. Read counts were normalized to relative abundance before their incorporation into downstream modeling workflows. MCMMs were constrained using metagenomic relative abundance profiles for all baseline samples using MICOM (v0.37.0), a framework for simulating microbial community metabolism^21^. Taxonomic profiles from baseline samples were mapped to genome-scale metabolic models (GEMs) from the AGORA database (v1.03)^36^. A relative abundance threshold of 0.001 was applied to exclude taxa representing less than 0.1% of the community composition.

For experimental group samples, probiotic supplementation was simulated *in silico* by modifying the baseline taxonomic composition. The relative abundances of pre-existing taxa were proportionally reduced to 95% of their original values, while five probiotic species from WBF-011 were introduced at a total relative abundance of 5% (1% each). The 5% supplementation level was selected to provide a detectable perturbation to the community while maintaining ecological realism.

For *Akkermansia muciniphiia* (AMUC), we used an updated species-level metabolic model from the AGORA2 database, as the AGORA1 version failed to predict growth under any simulated conditions.

### Model Growth

Community growth simulations were performed using MICOM’s cooperative tradeoff optimization, which balances individual taxon growth with overall community-wide metabolic efficiency. A tradeoff parameter of 0.99 was applied to ensure a balance between individual taxon viability and community-level metabolic fluxes. All models were supplied with a simulated growth medium reflective of an average European diet. In the absence of detailed dietary recall data, this can be used to approximate the average dietary consumption of individuals in North America, where this study took place. In the WBF-011 trial, inulin was included as an additional substrate in the medium to account for the prebiotic component of the synbiotic intervention. For expanded analysis in Arivale, inulin, pectin, starch, maltodextrin, cellulose (hemp seed), or arabinoxylan (psyllium husk), were added to the standard European diet to test prebiotic additions. Simulation using a high-fiber diet was also done. All dietary combinations were used to predict model outputs. Predicted growth rates for the five probiotic species were extracted from MICOM model outputs. To facilitate direct comparison with qPCR data in the WBF-011 trial, predicted growth rates were binarized using a threshold of 10^−3^ h^−1^. Growth rates below this value were classified as non-engraftment (0), while those above were classified as successful engraftment (1).

### qPCR Comparison

qPCR data collected from the original study was used as ground truth for the validation of model-based predictions. qPCR results failing quality control and filtering were removed. Inverse cycle threshold (1/Ct) was used as a proxy for species abundance. For each probiotic species, a low end threshold was defined by the mean value for 1/Ct for that species at Week 0. 1/Ct values from Week 12 were binarized around this threshold, for comparison with binarized MCMM-predicted growth rates.

### Statistical analysis

All statistical analysis conducted in this study was computed using Scipy (v 1.13.1)^41^

To assess the concordance between binarized MICOM-predicted engraftment and qPCR observations, the Cohen’s Kappa statistic was computed. This measure quantifies the level of agreement beyond what would be expected by chance, providing insight into the predictive accuracy of MCMMs for probiotic engraftment outcomes. Furthermore, Fisher’s exact test was used to evaluate whether the observed agreement between MICOM predictions and qPCR results in the confusion matrix was statistically significant.

Significance in differences in predicted butyrate and propionate between treatment groups in both the probiotic trial and the Arivale cohort (Fig. 3, Fig. 5) was computed using non-parametric Mann-Whitney U test. Significance in differences between predicted butyrate and propionate between optimal and standard of care interventions in the Arivale cohort (Fig. 6) was also determined using non-parametric Mann-Whitney U test.

Association between measured CRP values and predicted SCFA production was conducted using ordinary least squares linear regression. In the absence of available metadata, no confounding variables were used.

## Supplementary Material

1

## Data Availability

qPCR and metagenomic sequencing data for the trial by Perraudeau et al. 2020 are available upon request. Metagenomic sequencing data for the 156 members of the Arivale dataset are available on the NCBI Sequence Read Archive under accession number PRJNA1262070. All analysis conducted in the manuscript can be found at https://github.com/Gibbons-Lab/2024_probiotic_engraftment.
