# Supplementary material for: Metabolic modeling reveals determinants of synbiotic efficacy in a human intervention trial
Source: medRxiv. 2025 Jun 25:2025.06.24.25330246. Preprint. [Version 1] doi: 10.1101/2025.06.24.25330246 (PMC12262785; doi:10.1101/2025.06.24.25330246)
Supplement: 1 [file NIHPP2025.06.24.25330246V1-supplement-1.pdf]

medRxiv preprint doi: https://doi.org/10.1101/2025.06.24.25330246; this version posted June 25, 2025. The copyright holder for this preprint (which was not certified by peer review) is the author/funder, who has granted medRxiv a license to display the preprint in perpetuity.
It is made available under a CC-BY 4.0 International license .

Supplementary Figure Captions

medRxiv preprint doi: https://doi.org/10.1101/2025.06.24.25330246; this version posted June 25, 2025. The copyright holder for this preprint (which was not certified by peer review) is the author/funder, who has granted medRxiv a license to display the preprint in perpetuity.
It is made available under a CC-BY 4.0 International license .

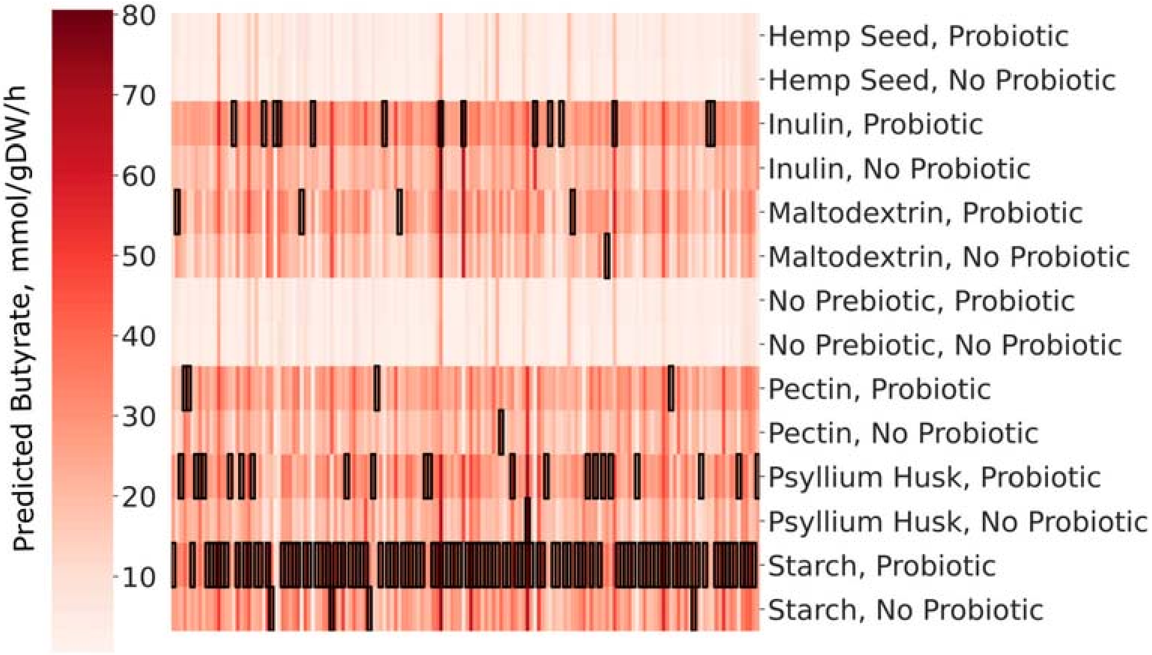

680
681 **Figure S1. Individual-specific optimal synbiotic treatments for maximizing butyrate
682 production**. MCMM-predicted butyrate production varied substantially across individuals (N =
683 156) in response to different synbiotic combinations. The combination of starch and the five-
684 strain probiotic cocktail produced the highest butyrate flux in the greatest number of
685 individuals. However, every other prebiotic/probiotic combination was optimal for at least one
686 individual, with the exceptions of: (1) hemp seed, (2) the no-probiotic condition, and (3) inulin
687 with no probiotic cocktail. Heatmap colors indicate predicted butyrate flux (mmol/gDW/h), and
688 black boxes denote the most effective treatment for each sample.

medRxiv preprint doi: https://doi.org/10.1101/2025.06.24.25330246; this version posted June 25, 2025. The copyright holder for this preprint (which was not certified by peer review) is the author/funder, who has granted medRxiv a license to display the preprint in perpetuity.
It is made available under a CC-BY 4.0 International license .

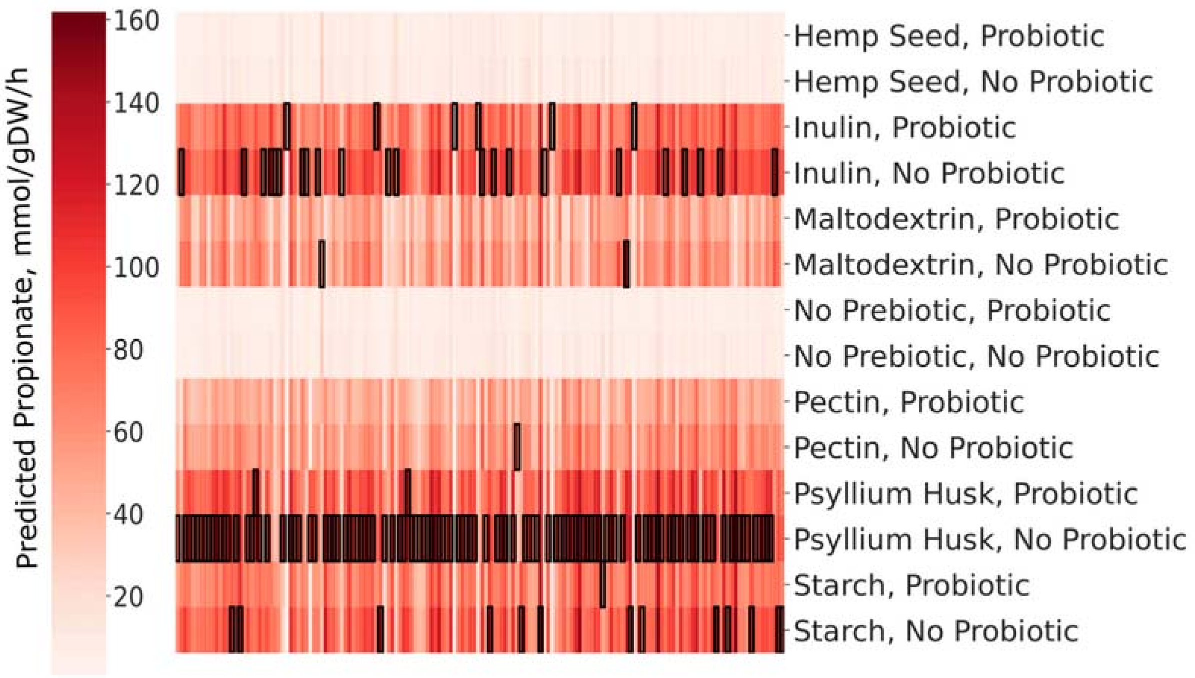

Figure S2. Individual-specific optimal synbiotic treatments for maximizing propionate production. MCMM-predicted propionate production also varied substantially across individuals (N = 156) in response to different synbiotic combinations, though slightly less than for butyrate. The combination of psyllium husk without a probiotic cocktail produced the highest propionate flux in the greatest number of individuals, indicating that the presence of the probiotic strains biases communities away from propionate production, toward butyrate production. However, every other prebiotic/probiotic combination was optimal for at least one individual, with the exceptions of: (1) hemp seed, (2) the no-probiotic condition, (3) maltodextrin with the probiotic cocktail and (4) inulin with the probiotic cocktail. Heatmap colors indicate predicted butyrate flux (mmol/gDW/h), and black boxes denote the most effective treatment for each sample.
